# Supplementary material for: Sexually dimorphic renal expression of mouse Klotho is directed by a kidney-specific distal enhancer responsive to HNF1b
Source: Commun Biol. 2024 Sep 14;7:1142. doi: 10.1038/s42003-024-06855-6 (PMC11401919; doi:10.1038/s42003-024-06855-6)
Supplement: Supplementary file 2 — Supplementary Information [file 42003_2024_6855_MOESM2_ESM.pdf]

Supplementary Information

Sexually dimorphic renal expression of mouse *Klotho* is directed by a kidney-specific distal enhancer responsive to HNF1b

Jakub Jankowski[1, *], Hye Kyung Lee[1], Chengyu Liu[2], Julia Wilflingseder[3], Lothar Hennighausen[1]

1 Section of Genetics and Physiology, Laboratory of Cellular and Molecular Biology, National Institute of Diabetes and Digestive and Kidney Diseases, US National Institutes of Health, Bethesda, Maryland, 20892, USA

2 Transgenic Core, National Heart, Lung, and Blood Institute, US National Institutes of Health, Bethesda, MD 20892, USA

3 Department of Physiology and Pathophysiology, University of Veterinary Medicine Vienna, Veterinärplatz 1, 1210 Vienna, Austria

Supplementary Figure 1

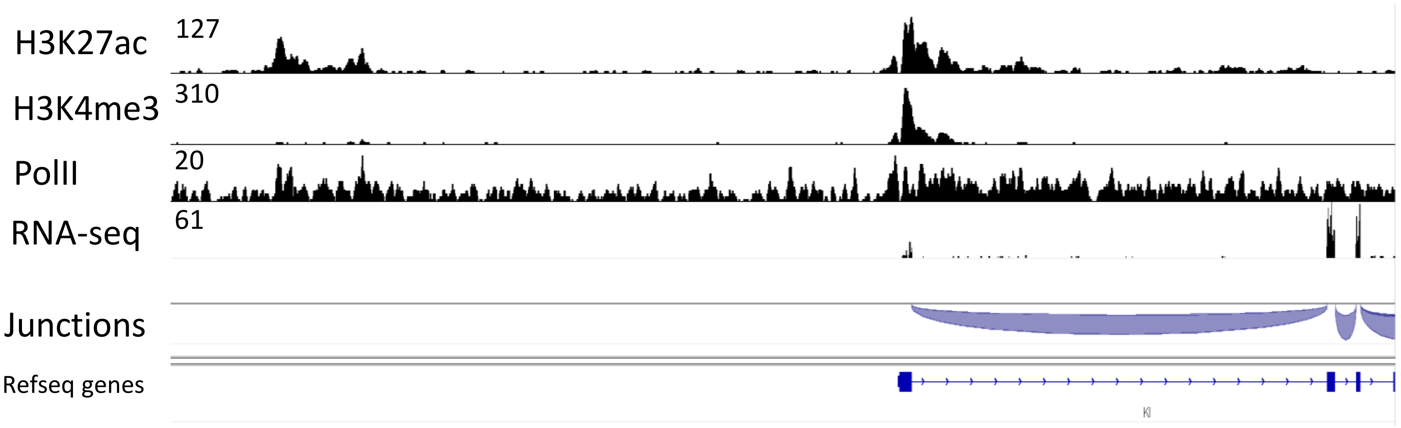

Supplementary Fig. 1 No eRNAs at are detected at the *Klotho* enhancer locus. ChIP-seq and RNA-seq were performed as described in the materials and methods section. Alignment of the RNA-seq to the PolII tracks indicates absence of enhancer RNA.

Supplementary Figure 2

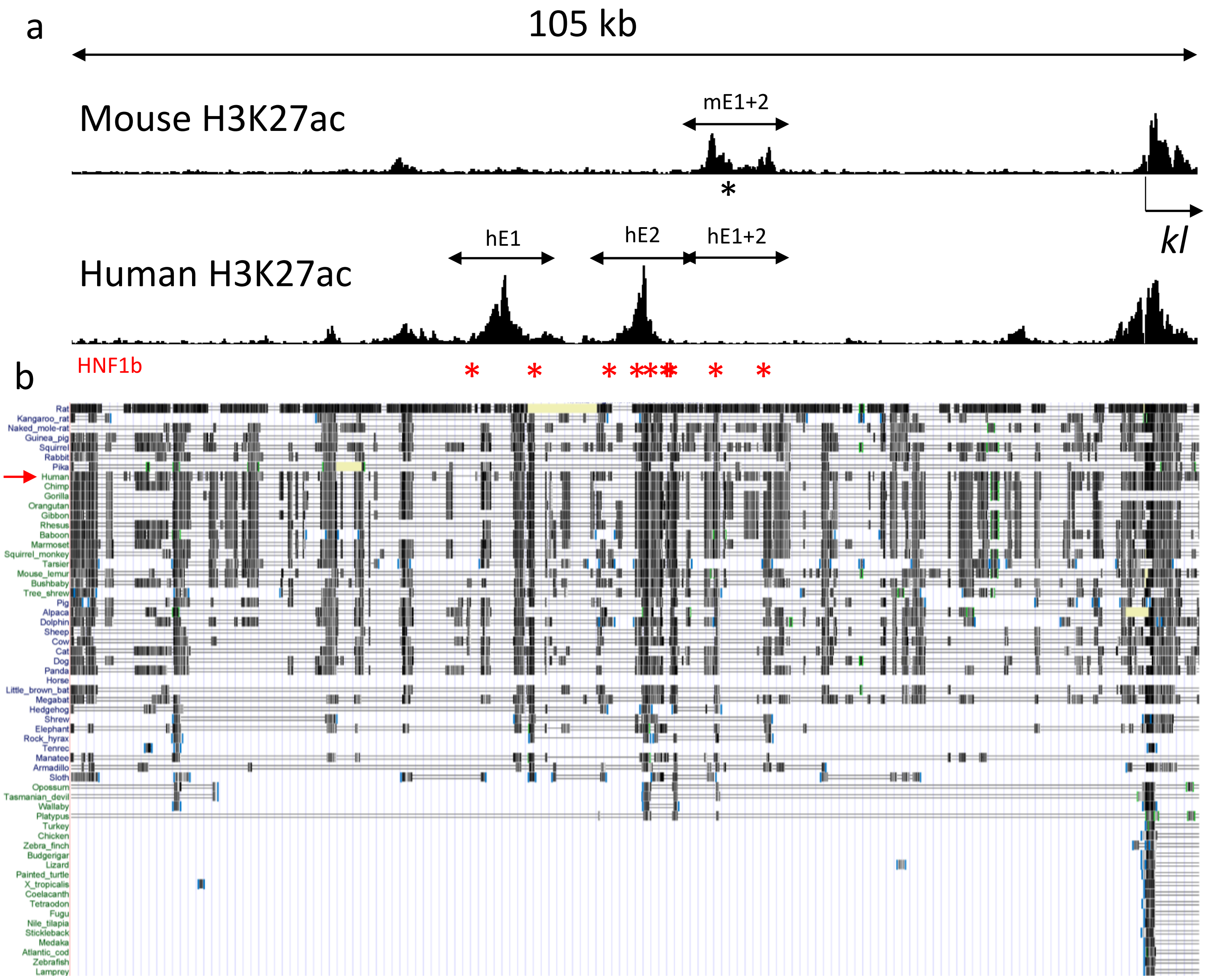

Supplementary Fig. 2 Klotho enhancers are conserved elements. Alignment human and mouse H3K27ac marks within 100kb region upstream of *Klotho* (a)**.** 10kb regions covering mouse enhancers E1 and E2 (mE1+2), analogous human region (hE1+2), as well as two putative human regulatory elements (hE1, hE2) were marked. Black asterisk – HNF1b site within mouse E1. Red asterisks – potential HNF1b motifs within human regions of interest. Conservation analysis graph of the 100kb region upstream of *Klotho*, relative to mouse sequence (b).

Supplementary Figure 3

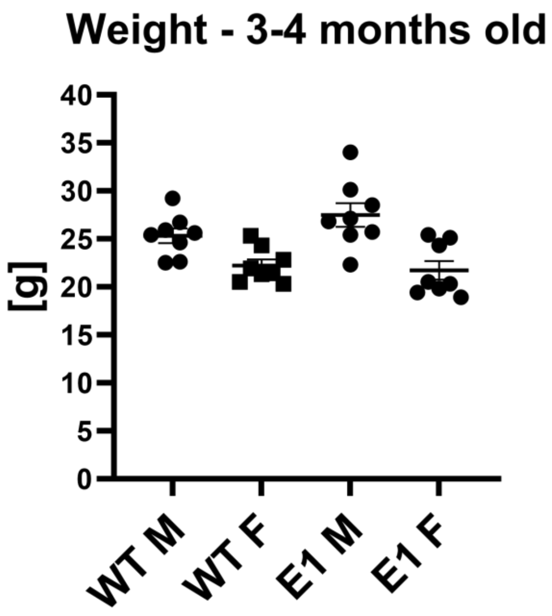

Supplementary Fig. 3 Mouse weight is not impacted by enhancer 1 knockout. Random litters of male, female, WT and E1 KO mice (n=8) at 3-4 months old were weighed. Unlike other Klotho knockout models, there is no significant difference in weight and mice survive into adultohood. Bar = SEM.

Supplementary Figure 4

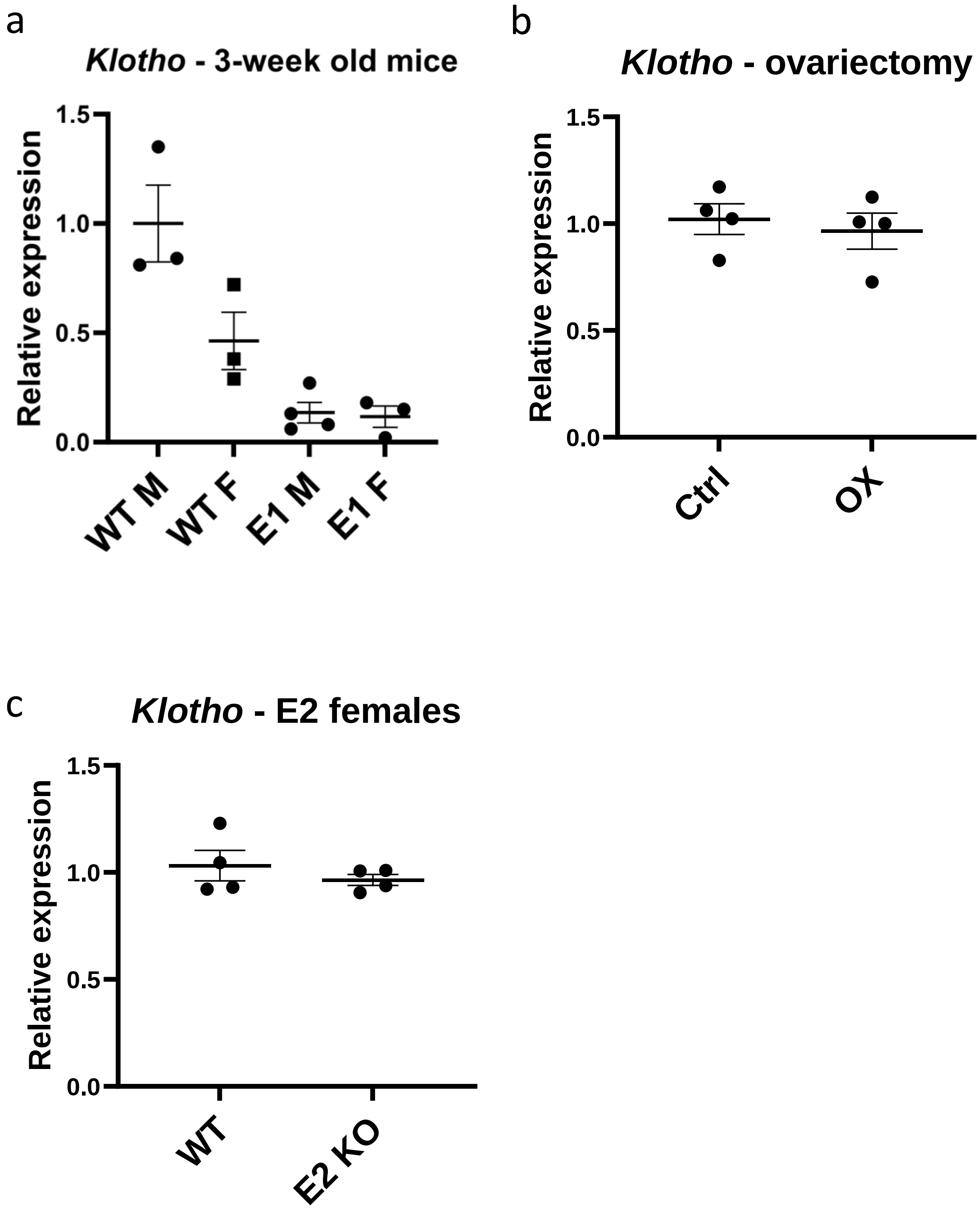

Supplementary Fig. 4 There is no indication of female sex hormones impacting Klotho levels. Renal Klotho mRNA was measured in 3-week old mice (A, n=4, data excluded due to undetectable *Klotho* expression) and in 8-week old mice ovariectomized at 3-weeks old (B, n=4). Sexual dimorphism of Klotho is detectable in prepubescent mice and ovariectomy does not impact mRNA levels. E2 deletion in females has no effect on Klotho expression (C, n=4.) Bar = SEM

Supplementary Figure 5

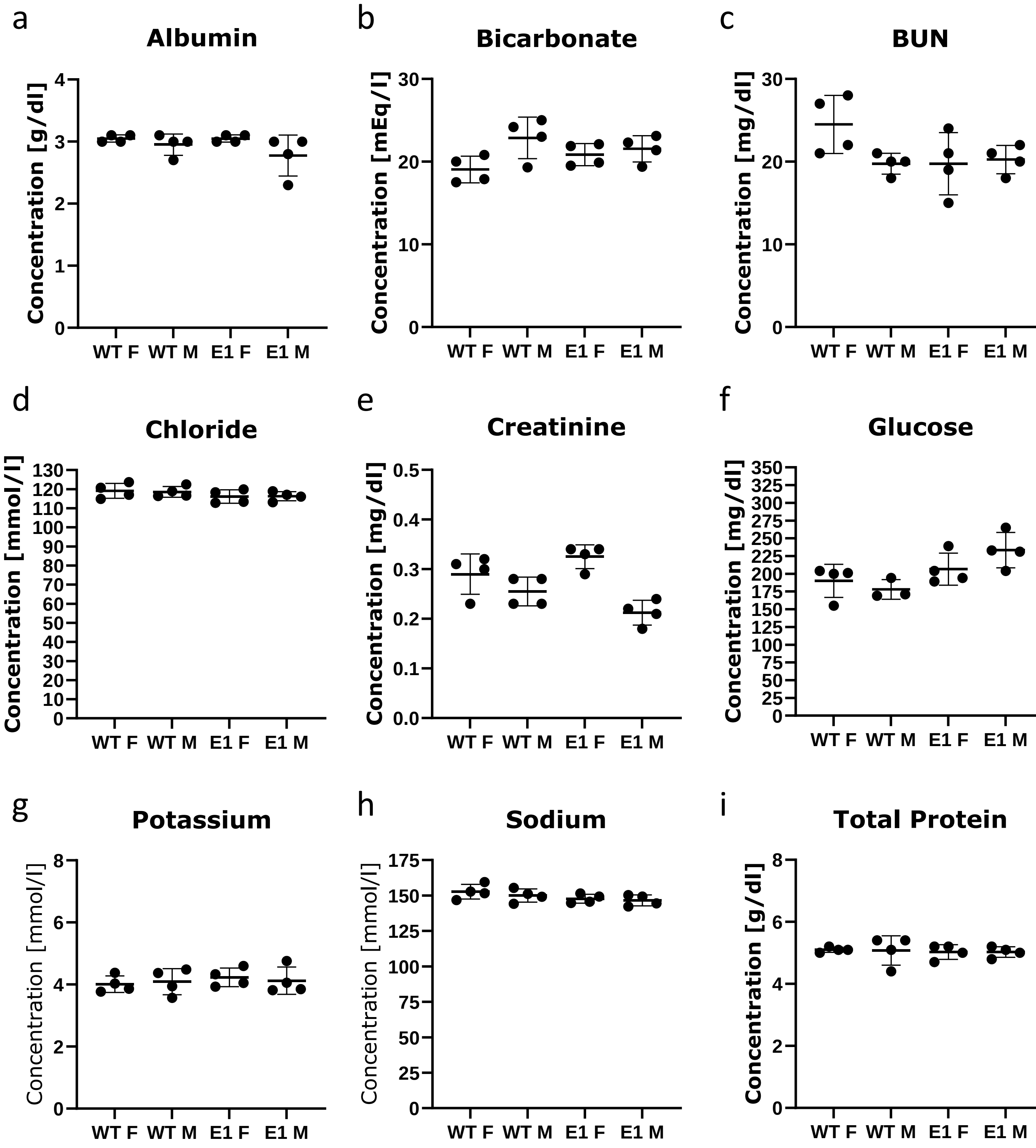

Supplementary Fig. 5 E1 deletion does not impact blood biochemistry. Serum biochemistry panel was performed as described in materials and methods section. No differences were observed between any of the experimental groups. A-I – n=4. Bar = SEM

Supplementary Figure 6

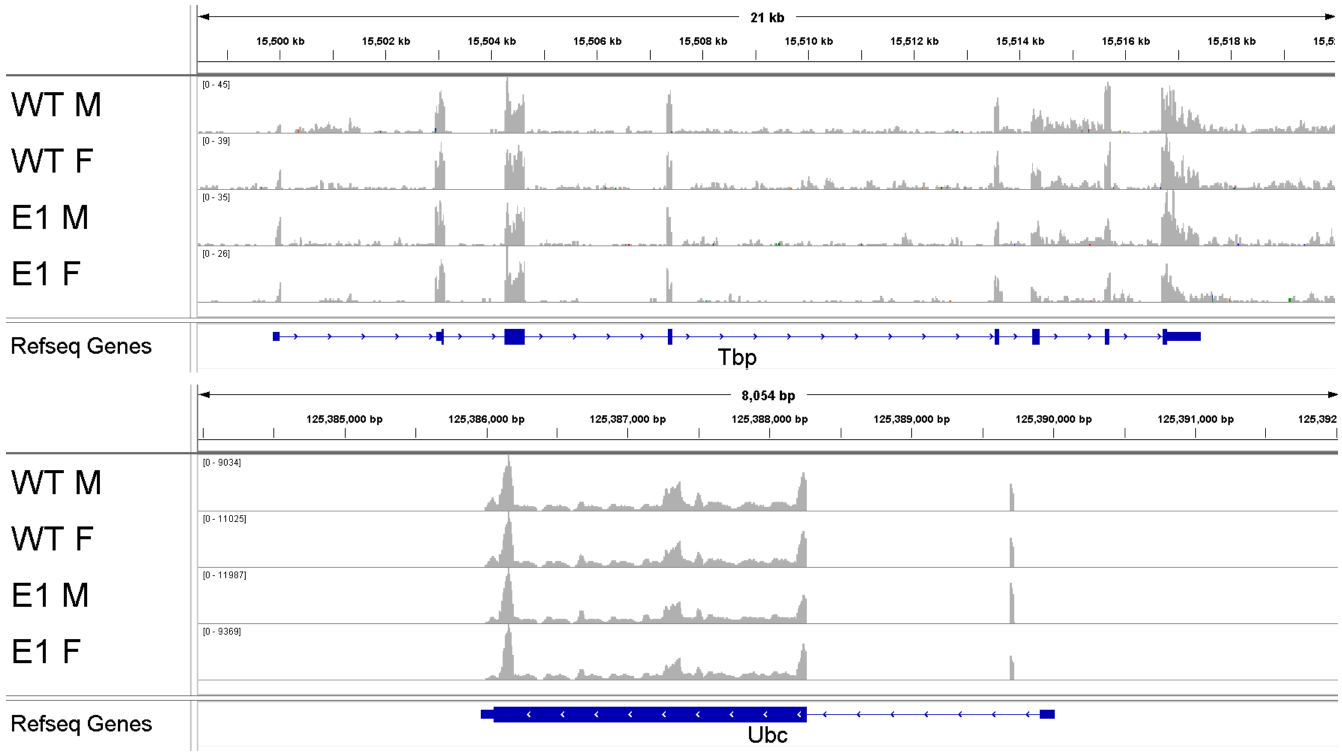

Supplementary Fig. 6 Control loci for RNA-seq analysis; housekeeping genes expressed in the kidney, not directly related to Klotho and displaying similar expression levels across genotypes.

Supplementary Figure 7

a b

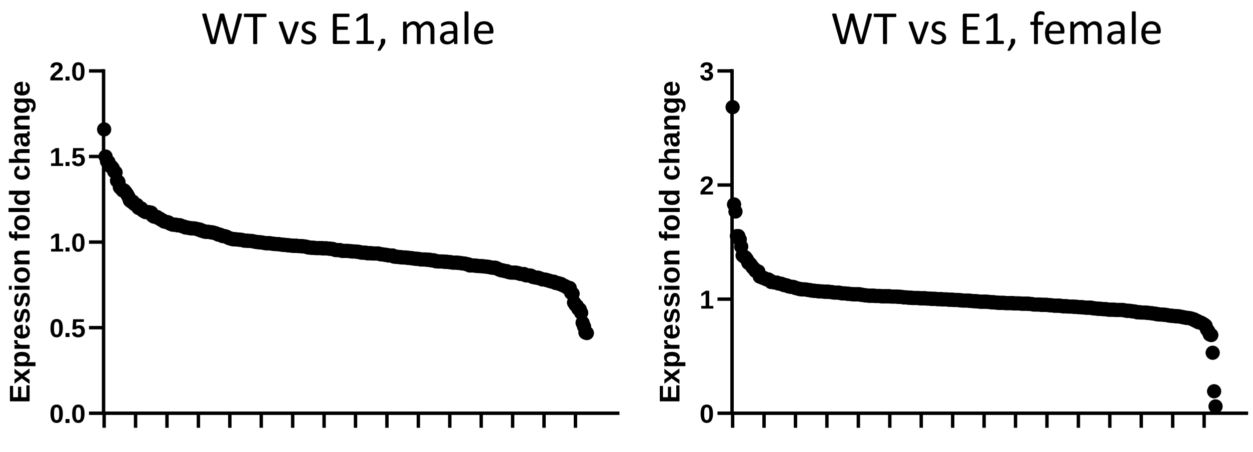

c

Gene	WT M	WT F	E1 M	E1 F	Fold change (male)
Slc14a2	2076	2409	1252	2223	1.66
Slco1a4	118	92	78	91	1.50
Slc9a9	83	98	57	77	1.47
Slc28a2	26	35	18	22	1.47
Slc14a1	133	210	92	219	1.45
Slc25a42	938	815	1596	923	0.59
Slc22a27	3	57	5	60	0.53
Slc16a14	1546	1310	3044	1513	0.51
Slc25a25	1790	2623	3794	2439	0.47
Slc7a12	84	6598	180	6852	0.47

d

Gene	WT M	WT F	E1 M	E1 F	Fold change (female)
Cacna1h	48	74	36	28	2.68
Cacna1e	41	46	33	25	1.83
Slc4a10	16	30	15	17	1.77
Cacna1c	155	274	156	176	1.55
Slc28a2	26	35	18	22	1.55
Slc22a28	3050	324	3030	468	0.69
Slc22a12	31487	11394	44739	16574	0.69
Slc6a7	11	19	14	35	0.53
Slc7a13	26343	63	28185	324	0.19
Slco1a1	19924	10	16351	159	0.06

Supplementary Fig. 7 Fold change in ion channel expression between male and female WT and E1 mice. Comparison of male (a) and female (b) expression of *SLC*, *TRPV* and *CNC* channels in mouse kidney. Five most up- and downregulated genes in each category are described in the tables (c, d). Only genes expressed in the kidney are represented (average read count >20 in all experimental groups, total of 339 genes).

Supplementary Figure 8

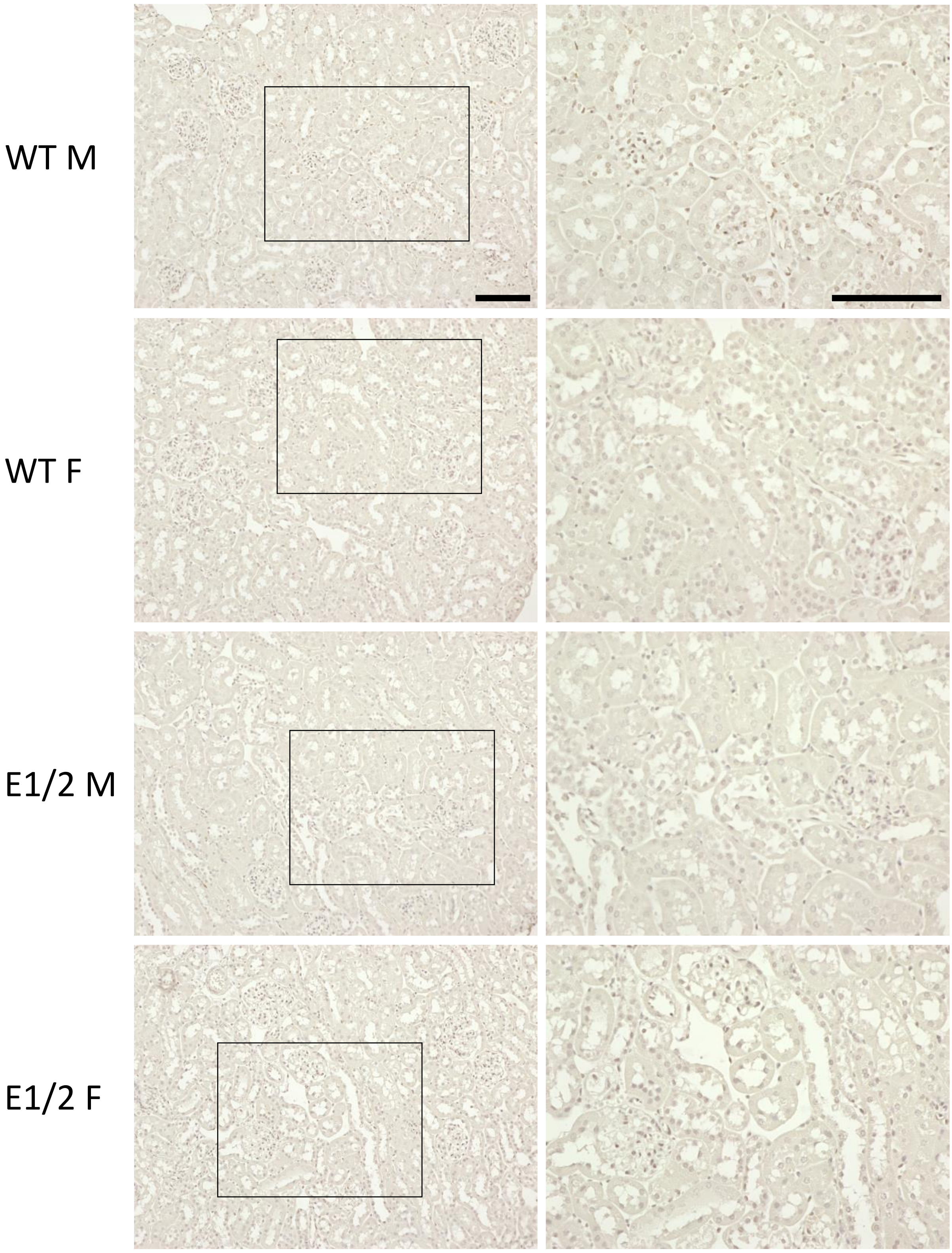

Supplementary Fig. 8 Negative control of renal klotho protein staining. Representative photographs of male and female renal tissue of WT and E1/2 knockouts exposed only to secondary antibody. Left: 200x magnification, right: outlined regions, 400x magnification, bar = 50 µm.

Supplementary Figure 9

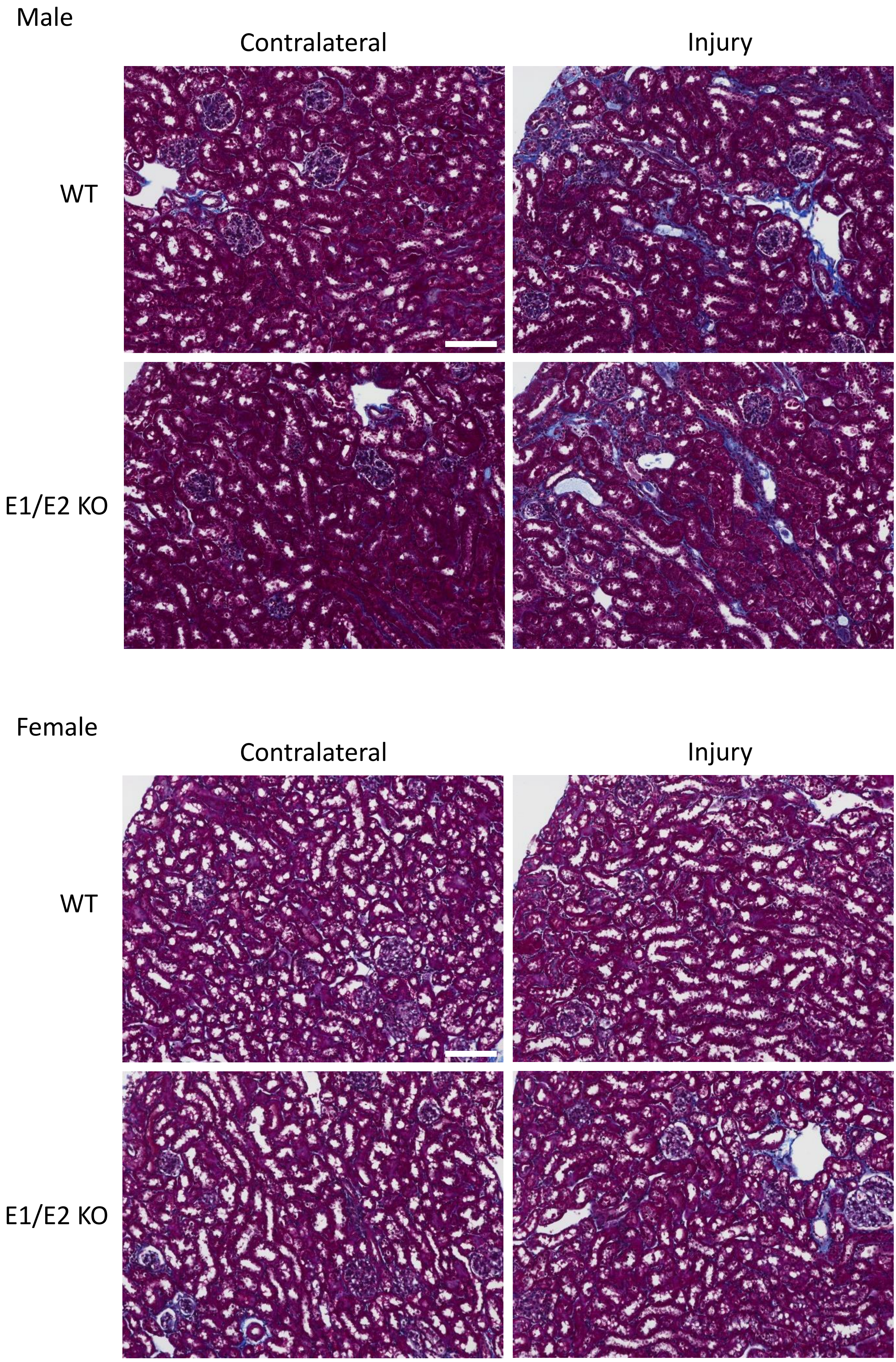

Supplementary Fig. 9 Representative photographs of injured kidneys and contralateral controls 28 days after unilateral kidney ischemia reperfusion surgery in male and female WT and E1/E2 mice. Bar = 50 μm.

Supplementary Table 1

	Sequence
E1 sgRNA	CTTCTTTCAGTGTGTCGCTTAAA
E2 sgRNA 1	ACCCTGATGCACCTCTGAAG
E2 sgRNA 2	TATCAGGTGGGATGAAGCCA
E1 Forward Primer 1	GAACTCAGAAATCTGCCTGCTCC
E1 Reverse Primer 1	GAGTAGCTGGGACTATGGTAATGC
E2 Forward Primer	CTATTCAGTGGTGTCGTTGGTCC
E2 Reverse Primer	TTGTTCTGTCCACCATCTCTTCCC

Supplementary Table 1 sgRNA and primer sequences used to construct and genotype E1, E2 and E1/E2 mice.

Supplementary Table 2

Mouse strain	Deletion sequence
E1 KO (1744 bp del)	CTTAACTCATCTGTTGATGGACTGTTATCCTGGCTGTTGAGAACAAAGCTTCAATAAATATGGGAGTGAAACTGCCCTTTG<br>ATGTACTGAGTTATTTTCCTTTGGGACTGGGAATTGCTGGATCACATGGAAATTTGTTCTTCATATTCTTGGTGACTGCACT<br>GGTTTCCATAATGACAGGACTAATATTAAGTCATGGTTTGAAGACAGCAGTCATCTTAGAGGTGTGTGGCCCTAGCACCC<br>TTCCCTTCCTCATGTGTAGCCCTAGGAAAGCAAACAAATAGTACAGGCTTCCCTATATTAGAAAGACCACAGCTGGGCTG<br>GAGAGATGGCTCAGTGGTTAAGAGCATTAACTGCTCTTCCAAAGGTCCTAAGTTCAAATCCCAGCAACCACATGGTGGCT<br>CACAACCATCTGTAATGAGATCTGATGCCCTCTTCTGGTGTGTCTGAAGACAGCTACAGTGTATTTACATATAATAAATAA<br>ATCTTAAAAAAAAAAAAAGAAAAAAAAAAAGACCATGGTTGTGTACTCTCTCACCTCCACAAGCCATGAGGATCAAAGGA<br>GACAAACTGGGAAGACATGCTGGCAACTCAGTCACTCTGTGAATGTTTGCTGTTCTCTCAGATTGGACCTTACTCTGTATC<br>TATCAAATTTGATATCTCCAGCAAGCCTAGTGAGTGTGGTTTGTCACTTAAGAACAGACTTAATTGGGGGGGGGGCTAAA<br>TCTGGGAGTGTTAGTACTAAACATGTCTCTATGTGACTGTCTCACCAGGTCCAGGAATAATTACAAGTTTAGGGGTTGAG<br>ATTTACTTGCATAGAGAGGGTAGGGATAGGGTGGGAAGGGTAAGAGGTTAGAAGGAAGGCAAAGGGTCACATGATGT<br>CAAGTGCTGGAGAACACTGGGCAGTGTTCAGTGTGGATGGAGAGGAAGAAGTCAGGGGAATCACAGCACCCCATTCAC<br>AGCTGAGCATCAGCTGGGTCCCGTGTCTGTGCTTGAGAGAGAGTCAGTAGTCAGTAGTCCTGGTGATAGGAGATGAGCT<br>GCAGACAGAGGTGAGATACTCCCTGGCTGGCTCTCCTGCCTGAGTGCTCTCTGCACTTGCAGCCTCACGACTTTCCCTTGT<br>ACTCTTGCACATGGCCATGGTAAAGGTTAACTGTGACCACCTGTCAAAGCCTGTTATATGTAGTTCCTCTCAGCCAGGCCT<br>TATGTGAAGCCTGGGTGACTGCAGGAGGCAAAGCAGACATGGCCCCTGGACTCTTGGCACTCAGTGTCTGTGGGGGAG<br>GTACACATGAAGACACAAGGAAATGCATACCCTAGAGCCAGGACACGTTGTGGAGGAGATGCCTGGAATCCTTGAGAGT<br>TTAGAGACAGGTCTGCACACTGAAGGTGTGTGAAATAAAAATAAACAAACACACAAGAGCGGTACATTAAATTAAAAAA<br>AAAATCTTGGCAAATACAAAGTGACTAGTTCTCACAGCAAGATCAAGTCTTGCTTTTAACTGAAGATGTTGGGGTTTTATT<br>GTTTTAACAAATATGTGTGTTTTCCTCTTTCCGAGTTTGGAAAAACAATCAAGCAGACATCCCACAAAGTGCAGCCCTGGA<br>AAACCTCGCCATCTGGACTGTCTGCTGGAAAAAACAGAAAGGTCACCCAGTGTGACTTGTCGAGCTTCACTAATTCCTGG<br>AATTCCGTAGTCATTTCTCCAAATTAATTTTTAACTTCTTTCAGTGTGTCGCTTAAAATAAAATTT
E1 1001 bp del	GTGTGTCGCTTAAAATAAAATTTTTATCTCACTAAGGGGGGAAAAACAGTGGGAGGGGAAAAAGGATGGAGGGGTTGA<br>CTTAACCTTTAAGTGTTAGCAGCACACAGAATTCAAAGTGCTTTACATAAATCAGAATCAGGGAAAAGCCAGGTGAGGTA<br>GTGCATTACCATAGTCCCAGCTACTCAGGAGACTGAGCAGGAAAACCCACCCCAGTTTCAAGGCCAGCCTGCGCTACAGA<br>GTGAGTTTAATGCTAGTCCAGGCTCTGCAGAGAGTTACTTTGTAACGGACTATAGAAATGATCCCTGAAAGTATAGTCTT<br>GAGAGTTATTTTGTATATTCCTCTTATCTTTTTTTTTTTTTTTTTTTTTTTTTTTTTTTTTTTGGTTTTTTTCGAGACAGGGTTTCTCT<br>GTATAGCCCTGGCTGTCCTGGAACTCACTTTGTAGACCAGGCTGGCCTCGAACTCAGATTCACCTGCCTCTGCCTCCCGAG<br>TGCTGGGATTAAAGGCGTGCACCACCACGCCCGGCCTTATTCCTCTTATCTTAGTATCTCCCAGAATTCCATGCCCCCTTAA<br>CTACAGGTAAAAGTGAGACTTATCTACAGTAAAGCTACAGTGTGAAGCTCCTGGGCTGTCTTTGTCAACTTCTTCTCCACT<br>CACATCATATTTGTTAATGAAACCTTTAGTGTTTATTCATTTATTTTATGTGTACAGATGCTTTTTGTTCTCTGGCATTTATAT<br>ATGTGCACCATATCCATACAGTGTCCATTGGGGCCAGGCAAGGGTGTCAGATCCTATGAAACTGATTAAGGACAGTTTTG<br>AGACATCCTGAGGGTGTTGGGAAGTGGACCCAGGTCCTCTGAAAGAGCAGCCAGTGCTTTTAACTGCTAAGCCATCTCTC<br>ATGCCCAGCCATGGTCTTTCTAGTACAGGGAAGCCTGTGATAGAAGCACCTTCTATAGAAAGTGTCCAGTGAGTGTAGCT<br>AGTGACTTCTAAGGTCAGTGGGTGTCTCA
E1 145 bp del	ATCTGGACTGTCTGCTGGAAAAAACAGAAAGGTCACCCAGTGTGACTTGTCGAGCTTCACTAATTCCTGGAATTCCGTAG<br>TCATTTCTCCAAATTAATTTTTAACTTCTTTCAGTGTGTCGCTTAAAATAAAATTTTTATCTCAC
E1 31 bp del	ATTTTTAACTTCTTTCAGTGTGTCGCTTAAA
E2 KO	ACCCTGATGCACCTCTGAAGAGGAGGTCAGAGAGTTCACAGGTAAAAGACGAGGTTGGAAATACTTTTAAAGAATACAA<br>GTTCACGGACCAGTGAGCCGCGGTTATGCATTTAGGAGCCGTCGCCTTTTGCGAGTGACAACTCACTGTCCACGTACTTG<br>GGCAGGAGAGATAGAAGTTAATAAATGAACACGAAGGGACCCAGAAAAGAGCATTGCTGATAGAACACAGCAGAGCTG<br>GGTGGATGCCAAACAGCCATGGGCACACCGCAGCGAAAGGAGGAACAACAGAAGCGCTTTCCAACAAATAACCAACAA<br>ATTTGGTGCGGAGGGCAGAGAGACGACTGCTGAAGGGAGCTGATCGTTGTGCTGGGCCAGGAGGATTAAGATGAGTTT<br>GCTGCTTATATCCAGACACTGTGCCATGTGCTCCCAGCTCGAGGGGGCTGAAGCCAGAACGCTGAGCAAGCCCAGCATG<br>ACATGTGCACAGACGTGGTCCCTCTCCCTCTGCAAGAGAAGCCCCAATGCCTTCCTGGTTCCAAATCGCCTGAAATTGAG<br>GTGTATGAAACTGGGTACTGGCTGGTAAATTTACCTTAGTAAGGCACGCTCTGAACTCCACTGCCGGTCAAAGGTGGATT<br>AATTCAGATGACCTGGAGGGAAAGTTCACACGAATCACCTCTCTCTCTCTCTCTCTCTCTCTCTCTCTCTCTCTCGCTC<br>TCGCTCTCGCTCTCTCGCTCTCTCTTGCTTTCTCTCTCTTGCTCTGTCTCTGTCTCTCTCTCTCTCTCGCTCTCTCGTTCTCTC<br>GCTCTCTCGCTCTCTCGCTCTCTCGCTCTCTCGCTCTCTCGCTCTCTCGCTCTCTCGCTCTCGCTCTCGTTCTCATCTCATCCC<br>ATGGCAGATGGGCGCACTCTGTTGTGGCTACAAAACTCAGGAACGCCCAGGCTTGCTAATTCAGGTATCAGGTGGGATG<br>AAGCCAAGGG
E1/E2 KO (E2)	CGAGGTTGGAAATACTTTTAAAGAATACAAGTTCACGGACCAGTGAGCCGCGGTTATGCATTTAGGAGCCGTCGCCTTTT<br>GCGAGTGACAACTCACTGTCCACGTACTTGGGCAGGAGAGATAGAAGTTAATAAATGAACACGAAGGGACCCAGAAAA<br>GAGCATTGCTGATAGAACACAGCAGAGCTGGGTGGATGCCAAACAGCCATGGGCACACCGCAGCGAAAGGAGGAACAA<br>CAGAAGCGCTTTCCAACAAATAACCAACAAATTTGGTGCGGAGGGCAGAGAGACGACTGCTGAAGGGAGCTGATCGTTG<br>TGCTGGGCCAGGAGGATTAAGATGAGTTTGCTGCTTATATCCAGACACTGTGCCATGTGCTCCCAGCTCGAGGGGGCTG<br>AAGCCAGAACGCTGAGCAAGCCCAGCATGACATGTGCACAGACGTGGTCCCTCTCCCTCTGCAAGAGAAGCCCCAATGC<br>CTTCCTGGTTCCAAATCGCCTGAAATTGAGGTGTATGAAACTGGGTACTGGCTGGTAAATTTACCTTAGTAAGGCACGCTC<br>TGAACTCCACTGCCGGTCAAAGGTGGATTAATTCAGATGACCTGGAGGGAAAGTTCACACGAATCACCTCTCTCTCTCT<br>CTCTCTCTCTCTCTCTCTCTCTCTCGCTCTCGCTCTCGCTCTCTCGCTCTCTCTTGCTTTCTCTCTCTTGCTCTGTCTCTGTCT<br>CTCTCTCTCTCTCTCGCTCTCTCGTTCTCTCGCTCTCTCGCTCTCTCGCTCTCTCGCTCTCTCGCTCTCTCGCTCTCTCGCTCTC<br>TCGCTCTCGCTCTCGTTCTCATCTCATCCCATGGCAGATGGGCGCACTCTGTTGTGGCT

Supplementary Table 2 Klotho E1 and E2 deletion sequences.

Supplementary Table 3

	Assay	Sample	Read count	Base total	Average read quality	Reads flagged as low quality
1	ChIP-seq	E1 F H3K4me3 read 1	67162543	3.3 Gbp	36	0
2	ChIP-seq	E1 F H3K4me3 read 2	67162543	3.3 Gbp	36	0
3	ChIP-seq	E1 F H3K27ac read 1	64115297	3.2 Gbp	36	0
4	ChIP-seq	E1 F H3K27ac read 2	64115297	3.2 Gbp	36	0
5	ChIP-seq	E1 M H3K4me3 read 1	14509762	725.4 Mbp	36	0
6	ChIP-seq	E1 M H3K4me3 read 2	14509763	725.4 Mbp	36	0
7	ChIP-seq	E1 M H3K27ac read 1	22246606	1.1 Gbp	36	0
8	ChIP-seq	E1 M H3K27ac read 2	22246607	1.1 Gbp	36	0
9	ChIP-seq	E1+2 M H3K4me3 read 1	28618044	1.4 Gbp	36	0
10	ChIP-seq	E1+2 M H3K4me3 read 2	28618045	1.4 Gbp	36	0
11	ChIP-seq	E1+2 M H3K27ac read 1	24002716	1.2 Gbp	36	0
12	ChIP-seq	E1+2 M H3K27ac read 2	24002717	1.2 Gbp	36	0
13	ChIP-seq	E2 M H3K4me3 read 1	26244819	1.3 Gbp	36	0
14	ChIP-seq	E2 M H3K4me3 read 2	26244820	1.3 Gbp	36	0
15	ChIP-seq	E2 M H3K27ac read 1	27816783	1.3 Gbp	36	0
16	ChIP-seq	E2 M H3K27ac read 2	27816784	1.3 Gbp	36	0
17	ChIP-seq	WT F H3K4me3 read 1	67702175	3.3 Gbp	36	0
18	ChIP-seq	WT F H3K4me3 read 2	67702176	3.3 Gbp	36	0
19	ChIP-seq	WT F H3K27ac read 1	62924546	3.1 Gbp	36	0
20	ChIP-seq	WT F H3K27ac read 2	62924547	3.1 Gbp	36	0
21	ChIP-seq	WT M H3K4me3 read 1	15227408	761.3 Mbp	36	0
22	ChIP-seq	WT M H3K4me3 read 2	15227409	761.3 Mbp	36	0
23	ChIP-seq	WT M H3K27ac read 1	13519717	675.9 Mbp	37	0
24	ChIP-seq	WT M H3K27ac read 2	13519717	675.9 Mbp	37	0
25	ChIP-seq	WT M HNF1b read 1	18685989	952.9 Mbp	39	0

	Assay	Sample	Read count	Base total	Average read quality	Reads flagged as low quality
1	RNA-seq	E1 F rep 1 read 1 lane 1	27003651	1.3 Gbp	37	0
2	RNA-seq	E1 F rep 1 read 2 lane 1	27003651	1.3 Gbp	37	0
3	RNA-seq	E1 F rep 1 read 1 lane 2	26881874	1.3 Gbp	37	0
4	RNA-seq	E1 F rep 1 read 2 lane 2	26881874	1.3 Gbp	37	0
5	RNA-seq	E1 F rep 2 read 1 lane 1	32797102	1.6 Gbp	37	0
6	RNA-seq	E1 F rep 2 read 2 lane 1	32797103	1.6 Gbp	37	0
7	RNA-seq	E1 F rep 2 read 1 lane 2	32757645	1.6 Gbp	37	0
8	RNA-seq	E1 F rep 2 read 2 lane 2	32757646	1.6 Gbp	37	0
9	RNA-seq	E1 F rep 3 read 1 lane 1	25269470	1.2 Gbp	37	0
10	RNA-seq	E1 F rep 3 read 2 lane 1	25269471	1.2 Gbp	37	0
11	RNA-seq	E1 F rep 3 read 1 lane 2	25083900	1.2 Gbp	37	0
12	RNA-seq	E1 F rep 3 read 2 lane 2	25083900	1.2 Gbp	37	0
13	RNA-seq	E1 F rep 4 read 1 lane 1	30996158	1.5 Gbp	37	0
14	RNA-seq	E1 F rep 4 read 2 lane 1	30996159	1.5 Gbp	37	0
15	RNA-seq	E1 F rep 4 read 1 lane 2	30794528	1.5 Gbp	37	0
16	RNA-seq	E1 F rep 4 read 2 lane 2	30794529	1.5 Gbp	37	0
17	RNA-seq	E1 M rep 1 read 1 lane 1	35047390	1.7 Gbp	37	0
18	RNA-seq	E1 M rep 1 read 2 lane 1	35047391	1.7 Gbp	37	0
19	RNA-seq	E1 M rep 1 read 1 lane 2	34958561	1.7 Gbp	37	0
20	RNA-seq	E1 M rep 1 read 2 lane 2	34958561	1.7 Gbp	37	0
21	RNA-seq	E1 M rep 2 read 1 lane 1	32138868	1.6 Gbp	37	0
22	RNA-seq	E1 M rep 2 read 2 lane 1	32138868	1.6 Gbp	37	0
23	RNA-seq	E1 M rep 2 read 1 lane 2	32116974	1.6 Gbp	37	0
24	RNA-seq	E1 M rep 2 read 2 lane 2	32116974	1.6 Gbp	37	0
25	RNA-seq	E1 M rep 3 read 1 lane 1	35020365	1.7 Gbp	37	0
26	RNA-seq	E1 M rep 3 read 2 lane 1	35020366	1.7 Gbp	37	0
27	RNA-seq	E1 M rep 3 read 1 lane 2	34959307	1.7 Gbp	37	0
28	RNA-seq	E1 M rep 3 read 2 lane 2	34959307	1.7 Gbp	37	0
29	RNA-seq	E1 M rep 4 read 1 lane 1	31548096	1.5 Gbp	37	0
30	RNA-seq	E1 M rep 4 read 2 lane 1	31548097	1.5 Gbp	37	0
31	RNA-seq	E1 M rep 4 read 1 lane 2	31418819	1.5 Gbp	37	0
32	RNA-seq	E1 M rep 4 read 2 lane 2	31418819	1.5 Gbp	37	0
33	RNA-seq	WT F rep 1 read 1 lane 1	34715443	1.7 Gbp	37	0
34	RNA-seq	WT F rep 1 read 2 lane 1	34715443	1.7 Gbp	37	0
35	RNA-seq	WT F rep 1 read 1 lane 2	34643522	1.7 Gbp	37	0
36	RNA-seq	WT F rep 1 read 2 lane 2	34643522	1.7 Gbp	37	0
37	RNA-seq	WT F rep 2 read 1 lane 1	31843298	1.5 Gbp	37	0
38	RNA-seq	WT F rep 2 read 2 lane 1	31843298	1.5 Gbp	37	0
39	RNA-seq	WT F rep 2 read 1 lane 2	31933257	1.5 Gbp	37	0
40	RNA-seq	WT F rep 2 read 2 lane 2	31933257	1.5 Gbp	37	0
41	RNA-seq	WT F rep 3 read 1 lane 1	31521892	1.5 Gbp	37	0
42	RNA-seq	WT F rep 3 read 2 lane 1	31521892	1.5 Gbp	37	0
43	RNA-seq	WT F rep 3 read 1 lane 2	31411780	1.5 Gbp	37	0
44	RNA-seq	WT F rep 3 read 2 lane 2	31411780	1.5 Gbp	37	0
45	RNA-seq	WT F rep 4 read 1 lane 1	31847111	1.5 Gbp	37	0
46	RNA-seq	WT F rep 4 read 2 lane 1	31847111	1.5 Gbp	37	0
47	RNA-seq	WT F rep 4 read 1 lane 2	31718935	1.5 Gbp	37	0
48	RNA-seq	WT F rep 4 read 2 lane 2	31718935	1.5 Gbp	37	0
49	RNA-seq	WT M rep 1 read 1 lane 1	29968427	1.4 Gbp	37	0
50	RNA-seq	WT M rep 1 read 2 lane 1	29968427	1.4 Gbp	37	0
51	RNA-seq	WT M rep 1 read 1 lane 2	29971507	1.4 Gbp	37	0
52	RNA-seq	WT M rep 1 read 2 lane 2	29971507	1.4 Gbp	37	0
53	RNA-seq	WT M rep 2 read 1 lane 1	33441905	1.6 Gbp	37	0
54	RNA-seq	WT M rep 2 read 2 lane 1	33441905	1.6 Gbp	37	0
55	RNA-seq	WT M rep 2 read 1 lane 2	33327171	1.6 Gbp	37	0
56	RNA-seq	WT M rep 2 read 2 lane 2	33327171	1.6 Gbp	37	0
57	RNA-seq	WT M rep 3 read 1 lane 1	34766658	1.7 Gbp	37	0
58	RNA-seq	WT M rep 3 read 2 lane 1	34766658	1.7 Gbp	37	0
59	RNA-seq	WT M rep 3 read 1 lane 2	34831510	1.7 Gbp	37	0
60	RNA-seq	WT M rep 3 read 2 lane 2	34831510	1.7 Gbp	37	0
61	RNA-seq	WT M rep 4 read 1 lane 1	32535439	1.6 Gbp	37	0
62	RNA-seq	WT M rep 4 read 2 lane 1	32535439	1.6 Gbp	37	0
63	RNA-seq	WT M rep 4 read 1 lane 2	32432937	1.6 Gbp	37	0
64	RNA-seq	WT M rep 4 read 2 lane 2	32432937	1.6 Gbp	37	0

Supplementary Table 3 Sequencing quality of submitted data.

Supplementary Table 4

Gene	WT M1	WT M2	WT M3	WT M4	WT F1	WT F2	WT F3	WT F4	E1 M1	E1 M2	E1 M3	E1 M4	E1 F1	E1 F2	E1 F3	E1 F4
N4bp2l2	2824	2116	1985	2146	2099	2272	2178	2500	2093	2265	2080	2219	2199	2035	2277	2527
Pds5b	1939	1659	1582	1565	1976	2008	1790	2058	1461	1432	1382	1738	1643	1796	1854	1937
Stard13	643	720	646	674	791	945	860	861	722	701	659	684	773	794	864	840
Vmn2r18	0	0	1	0	1	0	0	0	0	1	0	0	0	1	0	0

Supplementary Table 4 RNA-seq read counts of two genes flanking *Klotho* on either side does not reveal their regulation by enhancer deletion.
