# Supplementary material for: Sexually dimorphic renal expression of mouse Klotho is directed by a kidney-specific distal enhancer responsive to HNF1b
Source: Commun Biol. 2024 Sep 14;7:1142. doi: 10.1038/s42003-024-06855-6 (PMC11401919; doi:10.1038/s42003-024-06855-6)
Supplement: Supplementary file 3 — Description of Additional Supplementary File [file 42003_2024_6855_MOESM3_ESM.pdf]

Description of additional supplementary file

File name: Supplementary Data 1
Description: Gene counts and differentially expressed genes in WT and E1 KO male and female mice.

File name: Supplementary Data 2
Description: FIMO transcription motif analysis of human sequences upstream of Klotho gene.
