# Supplementary material for: Sexually dimorphic renal expression of mouse Klotho is directed by a kidney-specific distal enhancer responsive to HNF1b
Source: Commun Biol. 2024 Sep 14;7:1142. doi: 10.1038/s42003-024-06855-6 (PMC11401919; doi:10.1038/s42003-024-06855-6)
Supplement: Supplementary file 6 — Reporting Summary [file 42003_2024_6855_MOESM6_ESM.pdf]

nature portfolio

Corresponding author(s): Jakub Jankowski

Last updated by author(s): 8/13/2024

Reporting Summary

Nature Portfolio wishes to improve the reproducibility of the work that we publish. This form provides structure for consistency and transparency in reporting. For further information on Nature Portfolio policies, see our Editorial Policies and the Editorial Policy Checklist.

Statistics

For all statistical analyses, confirm that the following items are present in the figure legend, table legend, main text, or Methods section.

n/a	Confirmed	
☐	☒	The exact sample size (*n*) for each experimental group/condition, given as a discrete number and unit of measurement
☐	☒	A statement on whether measurements were taken from distinct samples or whether the same sample was measured repeatedly
☐	☒	The statistical test(s) used AND whether they are one- or two-sided<br>*Only common tests should be described solely by name; describe more complex techniques in the Methods section.*
☒	☐	A description of all covariates tested
☐	☒	A description of any assumptions or corrections, such as tests of normality and adjustment for multiple comparisons
☐	☒	A full description of the statistical parameters including central tendency (e.g. means) or other basic estimates (e.g. regression coefficient) AND variation (e.g. standard deviation) or associated estimates of uncertainty (e.g. confidence intervals)
☐	☒	For null hypothesis testing, the test statistic (e.g. *F*, *t*, *r*) with confidence intervals, effect sizes, degrees of freedom and *P* value noted<br>*Give P values as exact values whenever suitable.*
☒	☐	For Bayesian analysis, information on the choice of priors and Markov chain Monte Carlo settings
☒	☐	For hierarchical and complex designs, identification of the appropriate level for tests and full reporting of outcomes
☐	☒	Estimates of effect sizes (e.g. Cohen's *d*, Pearson's *r*), indicating how they were calculated

Our web collection on statistics for biologists contains articles on many of the points above.

Software and code

Policy information about availability of computer code

Data collection	Newly generated ChIP-seq and RNA-seq reads were collected using Illumina HiSeq 3000 and Novaseq 6000 hardware and software
Data analysis	ChIP-seq: QC analysis -FastQC (version 0.11.9); Quality filtering - Trimmomatic (version 0.36); Alignment - Bowtie2 (version 1.2.2), Samtools4 (version 1.8); Peak calling - MACS2 (version 2.2.7.1); Visualization -Integrative Genomics Viewer (version 2.16.2); Transcription factor motif detection - FIMO (version 5.4.1)<br>RNA-seq - QC, filtering and visualization as above, additionally: Alignment - RNA STAR (version 2.5.4a); Read count - HTSeq (version 0.9.1); Differential expression - DESeq2 (version 2.11.40.8)

For manuscripts utilizing custom algorithms or software that are central to the research but not yet described in published literature, software must be made available to editors and reviewers. We strongly encourage code deposition in a community repository (e.g. GitHub). See the Nature Portfolio guidelines for submitting code & software for further information.

Data

Policy information about availability of data

All manuscripts must include a data availability statement. This statement should provide the following information, where applicable:

- Accession codes, unique identifiers, or web links for publicly available datasets
- A description of any restrictions on data availability
- For clinical datasets or third party data, please ensure that the statement adheres to our policy

All ChIP-seq and RNA-seq datasets generated for this study were deposited in Gene Expression Omnibus (GEO) with the accession number GSE243946 (direct link: https://www.ncbi.nlm.nih.gov/geo/query/acc.cgi?acc=GSE243946).
In addition to original data, GEO series GSE114292 and GSE104907 were used to obtain H3K27ac, H3K4me3, PolII, GR and Esrry ChIP-seq data used in Figure 1a and b, and GSE129585 was used for human H3K27ac track in Supplementary Fig.S2. Further, data necessary to replicate figures, including original photographs, was deposited in Zenodo data sharing repository with the DOI: 10.5281/zenodo.12726086. Any additional data or materials are available on request.

Human research participants

Policy information about studies involving human research participants and Sex and Gender in Research.

Reporting on sex and gender	N/A
Population characteristics	N/A
Recruitment	N/A
Ethics oversight	N/A

Note that full information on the approval of the study protocol must also be provided in the manuscript.

Field-specific reporting

Please select the one below that is the best fit for your research. If you are not sure, read the appropriate sections before making your selection.

☒ Life sciences ☐ Behavioural & social sciences ☐ Ecological, evolutionary & environmental sciences

For a reference copy of the document with all sections, see nature.com/documents/nr-reporting-summary-flat.pdf

Life sciences study design

All studies must disclose on these points even when the disclosure is negative.

Sample size	Sample size was determined and restricted by animal space and ethics considerations, at the same time ensuring minimal sample size for appropriate statistical test (usually n >= 4).
Data exclusions	Only one experimental sample was excluded as noted in appropriate section (death during recovery from surgery procedure, postmortem tissue was not recovered).
Replication	Number of biological replicates is noted in every figure description. At least three replicates were used, excluding ChIP-seq, where only one replicate is provided, but derived from combined tissue from four animals to reduce variability.
Randomization	Animals were randomly selected for procedures with the age range and sex being only deciding factors.
Blinding	Surgeon was blinded to genotype, but not sex of the animals during all survival surgery procedures. Blinding was not applicable to other experiments.

Behavioural & social sciences study design

All studies must disclose on these points even when the disclosure is negative.

Study description	N/A
Research sample	N/A

Sampling strategy	N/A
Data collection	N/A
Timing	N/A
Data exclusions	N/A
Non-participation	N/A
Randomization	N/A

Ecological, evolutionary & environmental sciences study design

All studies must disclose on these points even when the disclosure is negative.

Study description	N/A
Research sample	N/A
Sampling strategy	N/A
Data collection	N/A
Timing and spatial scale	N/A
Data exclusions	N/A
Reproducibility	N/A
Randomization	N/A
Blinding	N/A

Did the study involve field work? ☐ Yes ☒ No

Field work, collection and transport

Field conditions	N/A
Location	N/A
Access & import/export	N/A
Disturbance	N/A

Reporting for specific materials, systems and methods

We require information from authors about some types of materials, experimental systems and methods used in many studies. Here, indicate whether each material, system or method listed is relevant to your study. If you are not sure if a list item applies to your research, read the appropriate section before selecting a response.

Materials & experimental systems

n/a	Involved in the study
☐	☒ Antibodies
☒	☐ Eukaryotic cell lines
☒	☐ Palaeontology and archaeology
☐	☒ Animals and other organisms
☒	☐ Clinical data
☒	☐ Dual use research of concern

Methods

n/a	Involved in the study
☐	☒ ChIP-seq
☒	☐ Flow cytometry
☒	☐ MRI-based neuroimaging

Antibodies

Antibodies used: H3K27ac (Abcam, ab4729), H3K4me3 (Millipore, 07-473), HNF1b (Invitrogen, 720259), Klotho (Cosmo Bio, KO603)

Validation: Per manufacturer's website:
H3K27ac (Abcam, ab4729) - 1912 references including PMID: 36163549, 36631623, 36536122, 36592610, 36632736
H3K4me3 (Millipore, 07-473) - 365 references, including PMID: 25845593, 25631790, 25567987, 25621826, 25402609
HNF1b (Invitrogen, 720259) - 2 references: PMID: 32636391, 35235779
Klotho (Cosmo Bio, KO603) - 3 references: PMID: 38665650, 24217253, 25037225

Eukaryotic cell lines

Policy information about cell lines and Sex and Gender in Research

Cell line source(s): N/A

Authentication: N/A

Mycoplasma contamination: N/A

Commonly misidentified lines (See ICLAC register): N/A

Palaeontology and Archaeology

Specimen provenance: N/A

Specimen deposition: N/A

Dating methods: N/A

☐ Tick this box to confirm that the raw and calibrated dates are available in the paper or in Supplementary Information.

Ethics oversight: N/A

Note that full information on the approval of the study protocol must also be provided in the manuscript.

Animals and other research organisms

Policy information about studies involving animals; ARRIVE guidelines recommended for reporting animal research, and Sex and Gender in Research

Laboratory animals: B6D2F1/J and C57BL/6N mice were obtained from Charles River. All animals used for experimental procedures were approximately 12-16 weeks old, except those presented in Supplementary Figure 3 a and b (3 and 8-weeks old).

Wild animals: NA

Reporting on sex: Sex of the experimental animals and their number is clearly stated in each figure description as the study describes sexually dimorphic gene regulation mechanism.

Field-collected samples: NA

Ethics oversight: All animals were handled according to the Guide for the Care and Use of Laboratory Animals (8th edition) and all animal experiments were approved by the Animal Care and Use Committee (ACUC) of National Institute of Diabetes and Digestive and Kidney Diseases (NIDDK, MD) and performed under the NIDDK animal protocol K089-LGP-20.

Note that full information on the approval of the study protocol must also be provided in the manuscript.

Clinical data

Policy information about clinical studies

All manuscripts should comply with the ICMJE guidelines for publication of clinical research and a completed CONSORT checklist must be included with all submissions.

Clinical trial registration	N/A
Study protocol	N/A
Data collection	N/A
Outcomes	N/A

Dual use research of concern

Policy information about dual use research of concern

Hazards

Could the accidental, deliberate or reckless misuse of agents or technologies generated in the work, or the application of information presented in the manuscript, pose a threat to:

No	Yes	
☒	☐	Public health
☒	☐	National security
☒	☐	Crops and/or livestock
☒	☐	Ecosystems
☒	☐	Any other significant area

Experiments of concern

Does the work involve any of these experiments of concern:

No	Yes	
☒	☐	Demonstrate how to render a vaccine ineffective
☒	☐	Confer resistance to therapeutically useful antibiotics or antiviral agents
☒	☐	Enhance the virulence of a pathogen or render a nonpathogen virulent
☒	☐	Increase transmissibility of a pathogen
☒	☐	Alter the host range of a pathogen
☒	☐	Enable evasion of diagnostic/detection modalities
☒	☐	Enable the weaponization of a biological agent or toxin
☒	☐	Any other potentially harmful combination of experiments and agents

ChIP-seq

Data deposition

☒ Confirm that both raw and final processed data have been deposited in a public database such as GEO.

☒ Confirm that you have deposited or provided access to graph files (e.g. BED files) for the called peaks.

Data access links
May remain private before publication.

GSE243946 (https://www.ncbi.nlm.nih.gov/geo/query/acc.cgi?acc=GSE243946)

Files in database submission

GSM7798772_E1_F_H3K27ac.bedgraph.gz
GSM7798772_E1_F_H3K27ac.tdf
GSM7798773_E1_F_H3K4me3.bedgraph.gz
GSM7798773_E1_F_H3K4me3.tdf
GSM7798774_E1_M_H3K27ac.bedgraph.gz
GSM7798774_E1_M_H3K27ac.tdf
GSM7798775_E1_M_H3K4me3.bedgraph.gz
GSM7798775_E1_M_H3K4me3.tdf
GSM7798776_E1+2_M_H3K27ac.bedgraph.gz
GSM7798776_E1+2_M_H3K27ac.tdf

GSM7798777_E1+2_M_H3K4me3.bedgraph.gz
GSM7798777_E1+2_M_H3K4me3.tdf
GSM7798778_E2_M_H3K27ac.bedgraph.gz
GSM7798778_E2_M_H3K27ac.tdf
GSM7798779_E2_M_H3K4me3.bedgraph.gz
GSM7798779_E2_M_H3K4me3.tdf
GSM7798780_WT_F_H3K27ac.bedgraph.gz
GSM7798780_WT_F_H3K27ac.tdf
GSM7798781_WT_F_H3K4me3.bedgraph.gz
GSM7798781_WT_F_H3K4me3.tdf
GSM7798782_WT_M_H3K27ac.bedgraph.gz
GSM7798782_WT_M_H3K27ac.tdf
GSM7798783_WT_M_H3K4me3.bedgraph.gz
GSM7798783_WT_M_H3K4me3.tdf
GSM8093064_WT_M_HNF1b.bedgraph.gz
GSM8093064_WT_M_HNF1b.tdf
JJ-7572_1_S1_L001_R2_001.fastq.gz
JJ-7572_2_S2_L001_R2_001.fastq.gz
JJ7748_1_2.fastq.gz
JJ7748_2_2.fastq.gz
JJ8839_1_1_S1_L001_R2_001.fastq.gz
JJ8839_1_2_S2_L001_R2_001.fastq.gz
JJ8839_1_9_S9_L001_R2_001.fastq.gz
JJ8839_1_10_S10_L001_R2_001.fastq.gz
JJ-7572_5_S5_L001_R2_001.fastq.gz
JJ-7572_6_S6_L001_R2_001.fastq.gz
JJ7748_5_2.fastq.gz
JJ7748_6_2.fastq.gz
JJ-7572_1_S1_L001_R1_001.fastq.gz
JJ-7572_2_S2_L001_R1_001.fastq.gz
JJ7748_1_1.fastq.gz
JJ7748_2_1.fastq.gz
JJ8839_1_1_S1_L001_R1_001.fastq.gz
JJ8839_1_2_S2_L001_R1_001.fastq.gz
JJ8839_1_9_S9_L001_R1_001.fastq.gz
JJ8839_1_10_S10_L001_R1_001.fastq.gz
JJ-7572_5_S5_L001_R1_001.fastq.gz
JJ-7572_6_S6_L001_R1_001.fastq.gz
JJ7748_5_1.fastq.gz
JJ7748_6_1.fastq.gz
kidney_Sham_Hnf1b_R_11082018_S55_L008_R1_001.fastq.gz

Genome browser session (e.g. UCSC)

https://genome.ucsc.edu/s/jankowskij7/Klotho%20enhancer%20KO%20review

Methodology

Replicates

One replicate pooling tissue from four animals was provided for each sample type.

Sequencing depth

Estimated >30 million reads per biological replicate.

Antibodies

H3K27ac (Abcam, ab4729), H3K4me3 (Millipore, 07-473), HNF1b (Invitrogen, 720259)

Peak calling parameters

MASC2 was used wit default parameters

Data quality

Quality was measured with FastQC and reads trimmed with Trimmomatic, followed by manual review of data quality such as identyfying peaks present independently of experimental intervention

Software

ChIP-seq: QC analysis -FastQC (version 0.11.9); Quality filtering - Trimmomatic (version 0.36); Alignment - Bowtie2 (version 1.2.2), Samtools4 (version 1.8); Peak calling - MACS2 (version 2.2.7.1); Visualization -Integrative Genomics Viewer (version 2.16.2)

Flow Cytometry

Plots

Confirm that:

☐ The axis labels state the marker and fluorochrome used (e.g. CD4-FITC).

☐ The axis scales are clearly visible. Include numbers along axes only for bottom left plot of group (a 'group' is an analysis of identical markers).

☐ All plots are contour plots with outliers or pseudocolor plots.

☐ A numerical value for number of cells or percentage (with statistics) is provided.

Methodology

Sample preparation	N/A
Instrument	N/A
Software	N/A
Cell population abundance	N/A
Gating strategy	N/A

☐ Tick this box to confirm that a figure exemplifying the gating strategy is provided in the Supplementary Information.

Magnetic resonance imaging

Experimental design

Design type	N/A
Design specifications	N/A
Behavioral performance measures	N/A

Acquisition

Imaging type(s)	N/A
Field strength	N/A
Sequence & imaging parameters	N/A
Area of acquisition	N/A

Diffusion MRI ☐ Used ☒ Not used

Preprocessing

Preprocessing software	N/A
Normalization	N/A
Normalization template	N/A
Noise and artifact removal	N/A
Volume censoring	N/A

Statistical modeling & inference

Model type and settings	N/A
Effect(s) tested	N/A

Specify type of analysis: ☐ Whole brain ☐ ROI-based ☐ Both

Statistic type for inference (See Eklund et al. 2016)	N/A
Correction	N/A

Models & analysis

n/a	Involved in the study
☒	☐ Functional and/or effective connectivity
☒	☐ Graph analysis
☒	☐ Multivariate modeling or predictive analysis

Functional and/or effective connectivity	N/A
Graph analysis	N/A
Multivariate modeling and predictive analysis	N/A
